# Challenges in Radiotherapy for Mandibular Cancer With Titanium Implant Reconstruction: A Case Report

**DOI:** 10.7759/cureus.100186

**Published:** 2025-12-27

**Authors:** Malak Chahid, Konimba Coulibaly, Assala Darragi Arfawi, Othmane Kaanouch, Fadila Kouhen

**Affiliations:** 1 Radiation Oncology, Cheikh Khalifa International University Hospital, Casablanca, MAR; 2 Radiation Oncology, Mohammed VI University of Sciences and Health (UM6SS), Casablanca, MAR; 3 Oral and Maxillofacial Surgery, Cheikh Khalifa International University Hospital, Casablanca, MAR; 4 Maxillofacial Surgery, Mohammed VI University of Sciences and Health (UM6SS), Casablanca, MAR; 5 Radiation Therapy, Laboratory of Sciences and Health Technologies, Higher Institute of Health Sciences, Hassan First University of Settat, Casablanca, MAR

**Keywords:** cancer surgery, radiation therapy (radiotherapy), squamous cell carcinoma of the head and neck, techniques, therapeutics

## Abstract

Mandibular cancer, a rare and aggressive malignancy, often requires extensive surgical resection followed by complex reconstruction. The advent of 3D-printed titanium implants has revolutionized mandibular reconstruction, offering precise anatomical restoration and improved functional outcomes. However, their integration into the radiotherapy workflow presents unique challenges, particularly in imaging and dose distribution.

This case report explores the management of a 71-year-old male with advanced mandibular squamous cell carcinoma, who underwent mandibular reconstruction using a custom 3D-printed titanium implant, followed by adjuvant radiotherapy. The challenges of imaging artifacts and dose perturbations caused by the titanium implant were addressed using advanced techniques, such as volumetric modulated arc therapy (VMAT) and manual artifact correction in the treatment planning system (TPS). A multidisciplinary approach, involving surgical, radiological, and radiotherapy teams, was crucial in optimizing treatment while minimizing damage to surrounding healthy tissues. This case underscores the importance of individualized treatment strategies and the need for ongoing research to refine protocols for integrating 3D-printed implants into radiotherapy. Ultimately, the combination of cutting-edge technology and collaborative care enhances patient outcomes and provides a model for managing complex head-and-neck oncology cases.

## Introduction

Mandibular cancer is a rare and aggressive malignancy that presents significant therapeutic challenges due to its impact on mastication, speech, and facial aesthetics, as well as its proximity to critical anatomical structures [1]. Treatment often involves extensive surgical resection, which can be debilitating and requires complex reconstruction to restore both function and aesthetics. The development of 3D printing technology has revolutionized mandibular reconstruction, allowing for the creation of highly customized titanium implants that precisely match the patient’s anatomy, thereby improving surgical outcomes and enhancing the patient’s quality of life [2,3]. However, the integration of these advanced reconstructive techniques presents unique obstacles in the context of adjuvant radiotherapy. The presence of a titanium implant can complicate radiation planning and delivery by affecting dose distribution, causing imaging artifacts, and making target volume delineation more difficult [4,5]. These challenges require careful, multidisciplinary planning to optimize radiotherapy while minimizing potential damage to surrounding healthy tissues.

Additionally, the intricate anatomy of the head and neck region poses further challenges, as critical structures such as the salivary glands, oral mucosa, and major neurovascular bundles, including the inferior alveolar nerve, lie in close proximity to the target area. Achieving a therapeutic balance between effective tumor control and minimizing radiation-induced toxicity to these vital structures requires advanced imaging modalities and precision radiotherapy techniques, such as intensity-modulated radiotherapy (IMRT) or proton therapy [6].

This case report aims to illustrate the radiotherapy planning challenges, dosimetric considerations, and clinical management of advanced mandibular squamous cell carcinoma treated with adjuvant radiotherapy following 3D-printed titanium implant reconstruction. It emphasizes the role of multidisciplinary collaboration, the integration of advanced radiotherapy planning technologies, and the importance of individualized treatment approaches to optimize outcomes in such challenging scenarios.

## Case presentation

A 71-year-old male presented with significant swelling of the left cheek, measuring approximately 15 cm in its greatest diameter, associated with intermittent bleeding from an ulcerative gingival lesion. His performance status was assessed at 0, indicating full activity without functional limitation. At presentation, the patient weighed 88 kg, with a body mass index (BMI) of 27 kg/m². He had no significant comorbidities, no history of smoking or alcohol consumption, and no known allergies.

Family history was negative for malignancies. The current illness began approximately six months prior to presentation, when the patient noticed a small lump in the left gingival region that progressively increased in size. Initially painless, the lesion became locally tender over time and was associated with intermittent bleeding. As the mass progressed, the patient reported difficulty chewing and mild dysphagia. Initial clinical examination revealed no palpable cervical lymphadenopathy.

Cervicofacial magnetic resonance imaging (MRI) demonstrated a locally advanced, expansive tumor centered on the horizontal and ascending ramus of the mandible, with extension to the submandibular gland and oropharynx. Cervical lymph node involvement was identified on imaging. Positron emission tomography-computed tomography (PET-CT) showed hypermetabolic activity in the gingival process, with invasion of adjacent soft tissues, including the pterygoid and masseter muscles, soft palate, and oropharynx, along with hypermetabolic ipsilateral cervical lymphadenopathy.

**Figure 1:**
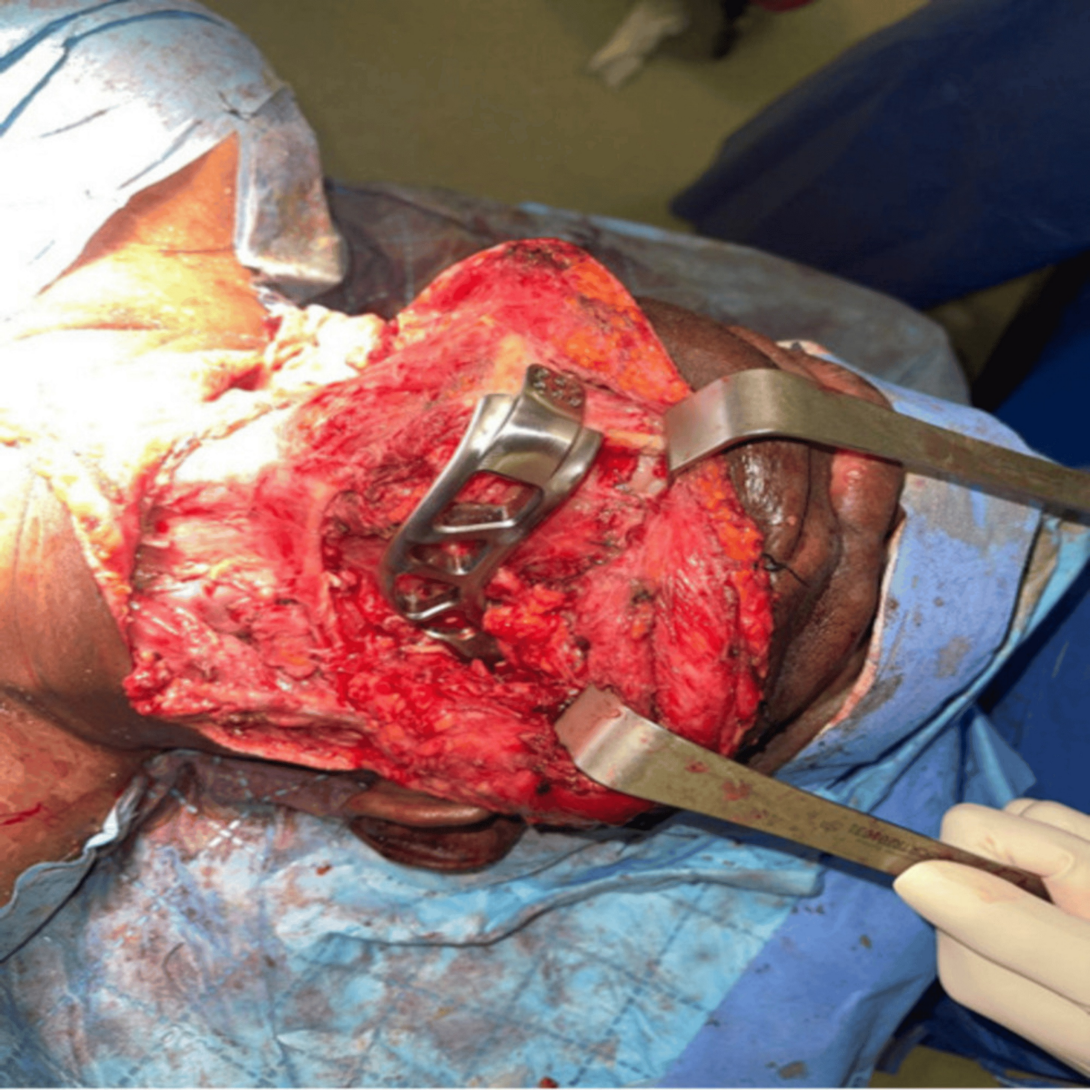
Intraoperative placement of the 3D-printed titanium implant in the mandibular defect

**Figure 2:**
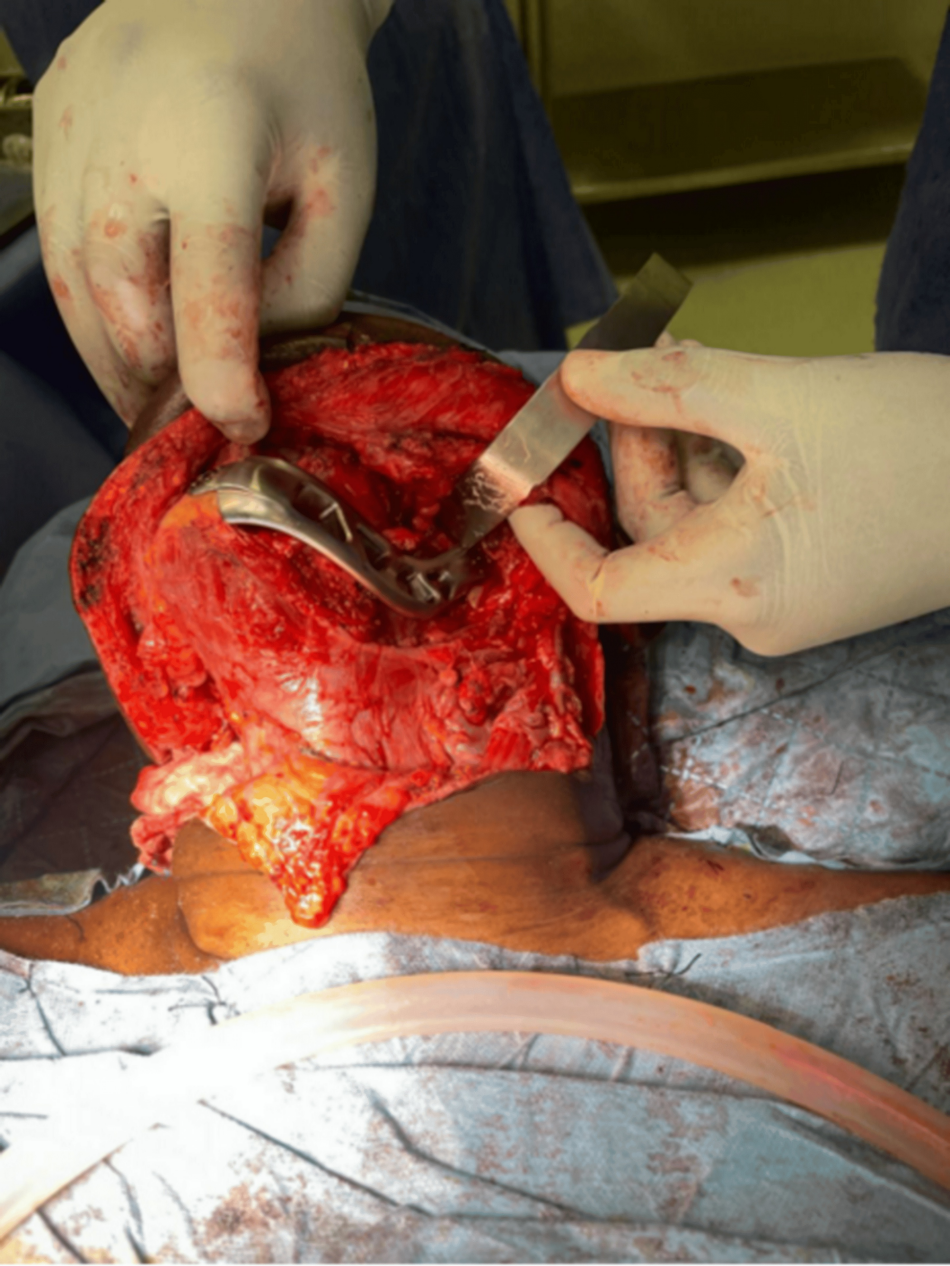
Intraoperative placement of the 3D-printed titanium implant in the mandibular defect

The patient received neoadjuvant chemotherapy consisting of three cycles of the TPF protocol (docetaxel, cisplatin, and fluorouracil), resulting in a favorable clinical response characterized by softening of the tumor mass. He subsequently underwent surgical resection of the left hemimandible, including the medial pterygoid and masseter muscles, along with cervical lymphadenectomy. Histopathological examination revealed moderately differentiated, keratinizing squamous cell carcinoma infiltrating the mandible and adjacent striated muscle. There was no evidence of perineural invasion or vascular emboli, and both anterior and posterior bone margins were clear. Two of 50 examined lymph nodes were positive for malignancy (2/50). Mandibular reconstruction was performed using a patient-specific 3D-printed titanium implant (Figures 1-2).

**Figure 3:**
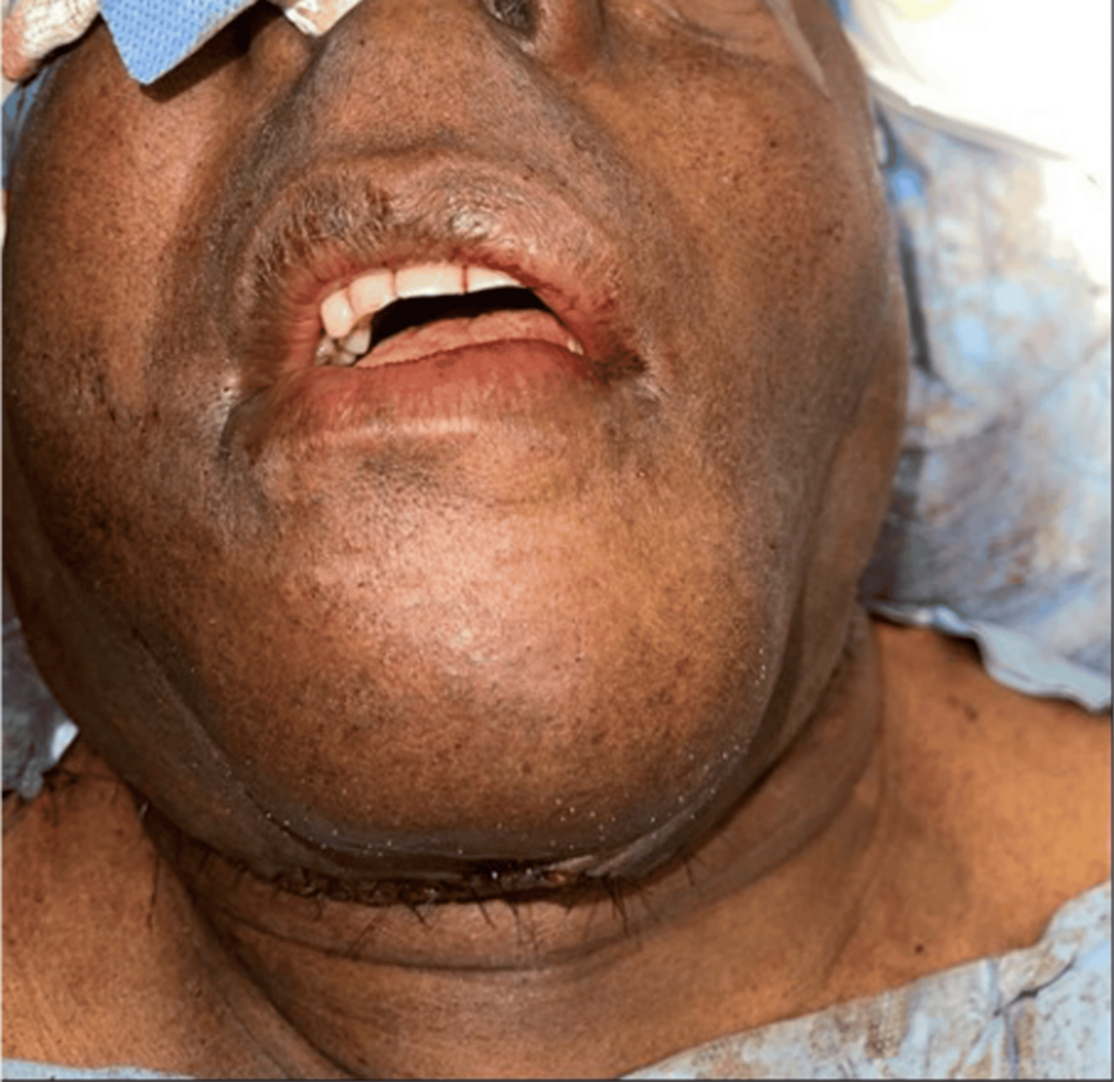
Postoperative front view of the patient showing the final aesthetic result after suture

This reconstructive approach aimed to restore mandibular continuity, structural integrity, and functional outcomes, including speech and mastication (Figure 3).

**Figure 4:**
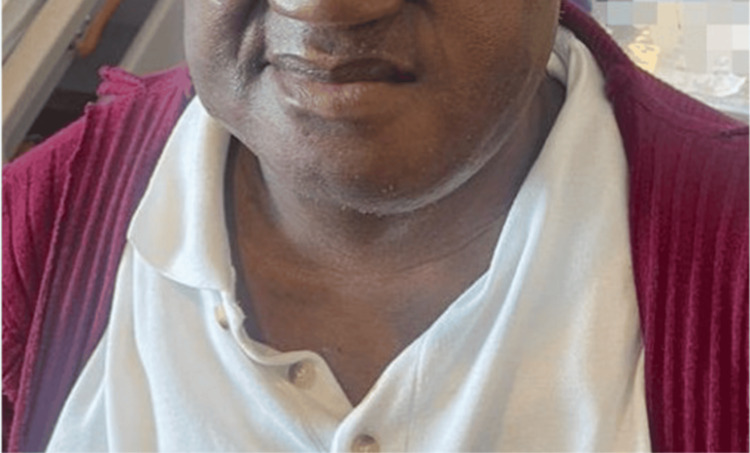
Postoperative photograph of the patient at 10 days post-surgery, showing the healing and scar tissue

The titanium implant provided stable mechanical support and facilitated postoperative recovery (Figure 4). The implant is clearly visualized and indicated by arrows on intraoperative and postoperative images.

Postoperative wound healing was delayed. This delay was considered multifactorial, and clinical evaluation with laboratory investigations did not reveal infection, metabolic abnormalities or other pathological causes of impaired healing. As a result, adjuvant radiotherapy was initially planned but postponed.

During the postoperative period, and prior to radiotherapy initiation, the patient received weekly cisplatin, which was well tolerated and resulted in clinical stabilization. He was later readmitted for management of recurrent cervical lymphadenopathy related to the previously treated squamous cell carcinoma. A gastrostomy was placed for nutritional support, and antibiotic therapy was initiated. Following multidisciplinary discussion, concurrent chemoradiotherapy was recommended, consisting of weekly cisplatin at a dose of 40 mg/m².

Radiotherapy was delivered using a 6 MV photon beam on a TrueBeam linear accelerator (Varian Medical Systems, Palo Alto, CA, USA), with the volumetric modulated arc therapy (VMAT) technique. Daily image guidance was performed using cone-beam computed tomography (CBCT). A total dose of 70 Gy was administered in 35 fractions of 2 Gy, five days per week.

The patient was followed through regular outpatient visits with clinical and radiological assessments. At three months post-radiotherapy, he demonstrated good local control, with no clinical or radiological evidence of residual or recurrent disease on MRI. The titanium implant remained well integrated, without signs of exposure or mechanical failure. Swallowing function gradually improved, allowing partial oral intake in combination with enteral nutrition, and speech intelligibility was preserved.

At six months, PET-CT confirmed the absence of metabolic activity at the primary site and regional lymph nodes. The patient experienced mild xerostomia and grade 1 mucosal fibrosis, with no clinical or radiological evidence of osteoradionecrosis. Functional outcomes remained favorable, with progressive return to daily activities and no requirement for opioid analgesics. At nine months, the patient remained in clinical remission under continued multidisciplinary follow-up.

## Discussion

The use of patient-specific 3D-printed titanium implants for mandibular reconstruction represents a major advancement in surgical oncology, offering precise anatomical restoration and improved functional outcomes compared with conventional reconstructive techniques [7]. However, their integration into postoperative radiotherapy workflows can introduce specific technical challenges related to imaging accuracy and dose distribution [8,9].

**Figure 5:**
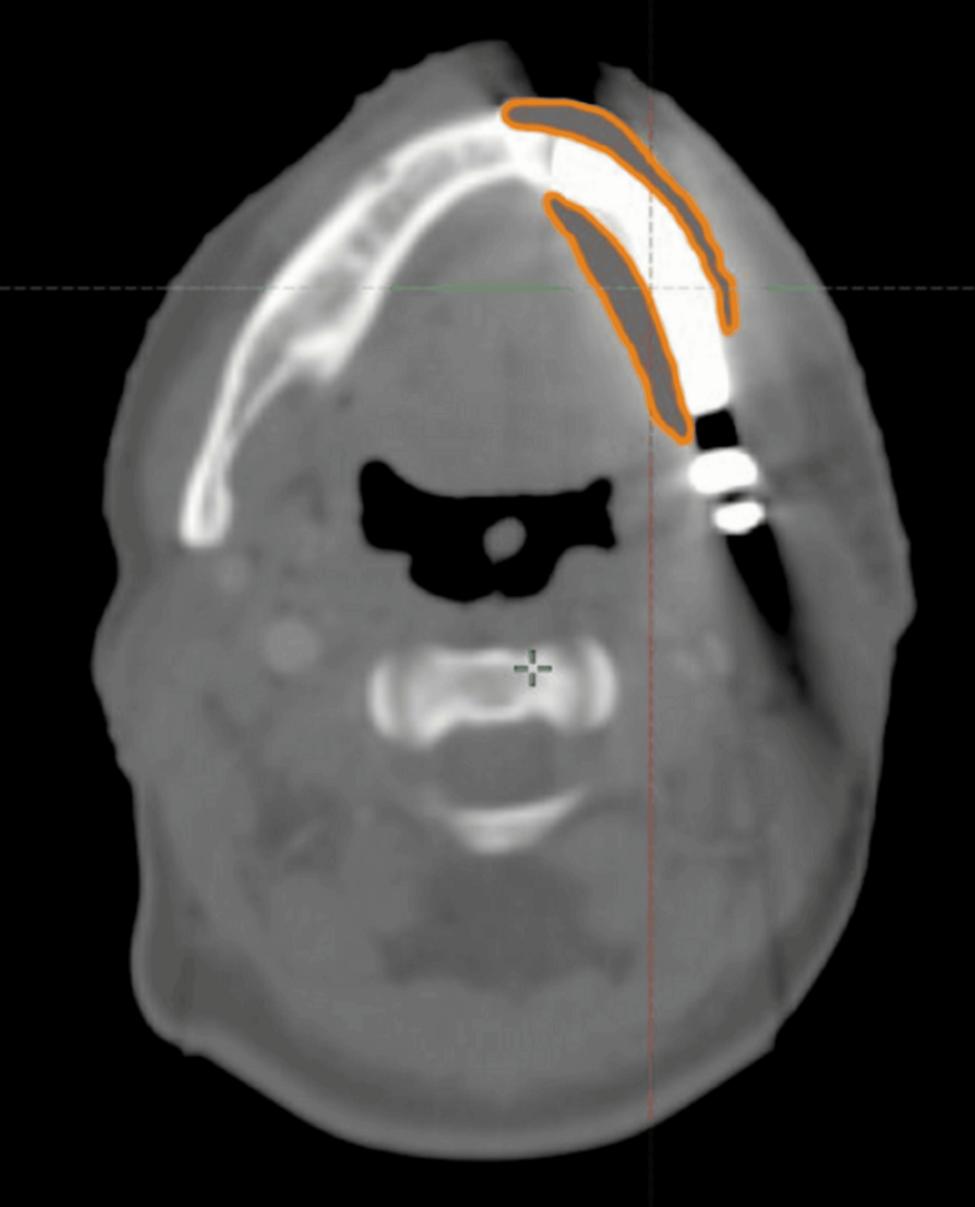
Scanographic image demonstrating artifact correction of the titanium implant using Hounsfield unit density adjustment

Titanium implants are known to generate imaging artifacts, primarily due to beam hardening and scatter effects on CT. These artifacts may obscure critical anatomical structures and complicate the accurate delineation of target volumes and organs at risk (OARs), particularly in the anatomically complex head and neck region [10]. In the present case, a pragmatic imaging strategy was adopted to address these limitations. Rather than relying on CT-MRI fusion for artifact correction, a manual density correction approach was applied: regions affected by metal-induced artifacts were contoured and assigned a soft-tissue-equivalent density. This method allowed the Anisotropic Analytical Algorithm (AAA) to perform dose calculations without overestimation or underestimation in artifact-prone areas, while preserving native Hounsfield unit (HU) values in unaffected regions (Figure 5).

The patient was treated using VMAT with a 6 MV photon beam delivered by a TrueBeam linear accelerator (Varian Medical Systems). VMAT was selected for its ability to deliver a highly conformal dose, allowing precise sculpting around the implant while minimizing radiation exposure to adjacent OARs. Dose calculations were performed using the AAA in the treatment planning system (TPS) Eclipse (Version 13.6), which incorporates heterogeneity corrections but remains sensitive to density inaccuracies in artifact-affected regions, while areas with naturally low HU values, which were not artifact-related, were left unaltered.

Titanium implants also impact radiotherapy dose distribution, primarily through attenuation and backscatter effects. Studies have shown that these implants can cause dose deviations of up to 7% in photon beams, which is clinically significant, particularly in the anatomically complex head and neck region [5,9]. In this context, VMAT was selected for its ability to deliver highly conformal dose distributions while minimizing exposure to OARs. Beam angles were carefully optimized to reduce direct irradiation of the titanium structure, while maintaining adequate tumor coverage. Additionally, in vivo dosimetry was performed using an electronic portal imaging device (EPID), allowing real-time verification of dose delivery at different angles. Analysis of portal images confirmed that dose deviations remained within acceptable clinical tolerances (<5%), validating the accuracy of AAA-based dose calculations following artifact corrections.

Current data on the radiotherapy implications of 3D-printed titanium implants remain limited, highlighting the need for dedicated research to establish optimized protocols. Future studies should explore strategies to mitigate imaging artifacts and dose perturbations, thereby enabling the seamless integration of these implants into comprehensive treatment plans [6,11].

This case underscores the critical role of a multidisciplinary approach in managing complex oncological scenarios. Collaboration among surgical, radiological, and radiotherapy teams was essential in addressing the unique challenges posed by the implant. This synergy allowed for the integration of advanced imaging and radiotherapy planning techniques, ensuring precise and effective treatment while preserving the integrity of both the implant and surrounding healthy tissues [4].

This report is limited by its single-case design, which restricts the generalizability of the findings. The observed dosimetric behavior may vary depending on implant geometry, location, and treatment technique. Additionally, conclusions regarding long-term oncologic outcomes cannot be drawn from a single observation, and larger prospective studies are required to establish standardized radiotherapy planning guidelines for patients reconstructed with 3D-printed titanium mandibular implants.

## Conclusions

While 3D-printed titanium implants have significantly expanded reconstructive options in mandibular oncology, their presence introduces specific challenges in radiotherapy planning and delivery. This case highlights the importance of tailored imaging strategies, advanced radiotherapy techniques, and close multidisciplinary collaboration to safely and effectively manage these challenges. Although conclusions regarding long-term oncologic outcomes cannot be drawn from a single case, this report illustrates practical approaches to mitigate imaging artifacts and dose perturbations associated with metallic implants. Further clinical experience and dedicated studies are needed to refine radiotherapy planning strategies and establish standardized protocols for patients reconstructed with 3D-printed titanium mandibular implants.
